# Generative AI–Assisted Progressive-Disclosure Case-Based Learning for Clinical Reasoning in Occupational Medicine: Quasi-Experimental Study

**DOI:** 10.2196/103584

**Published:** 2026-09-22

**Authors:** Peng Su, Min Hu, Chengzhi Chen, Shangcheng Xu

**Affiliations:** 1 Department of Occupational and Environmental Health School of Public Health Chongqing Medical University Chongqing, Chongqing China; 2 School of Business Chongqing College of Finance and Economics Chongqing, Chongqing China

**Keywords:** generative AI, case-based learning, clinical reasoning, occupational medicine, medical education

## Abstract

**Background:**

Case-based learning (CBL) promotes transfer of knowledge to practice, yet occupational health CBL must also develop exposure assessment and epidemiologic thinking. Static, single-session cases that disclose all information upfront can truncate iterative reasoning and encourage premature diagnostic closure. Generative AI (GenAI) can support the efficient development of high-fidelity, progressively disclosed cases, but hallucination risks require strict quality control.

**Objective:**

This study aimed to develop and evaluate a multicomponent GenAI-assisted, 4-act progressive-disclosure CBL package for an occupational lead poisoning module, using a human-in-the-loop workflow to mitigate hallucination risk.

**Methods:**

In a nonrandomized posttest controlled quasi-experimental study, 224 undergraduates were assigned by administrative class to an intervention group (n=114) or a control group (n=110). The control group received conventional static CBL; the intervention group received a GenAI-assisted progressive-disclosure CBL package. The primary outcome was the standardized individual case-analysis assignment score. Secondary outcomes were the delayed final examination score, 5 self-reported learning experience domains, and 3 video-derived behavioral engagement indicators. Subgroup analyses by academic major and group-by-major interaction tests were exploratory.

**Results:**

Baseline characteristics were comparable between groups. The intervention group scored higher on case analysis (mean 88.41, SD 3.85 vs mean 82.51, SD 3.94; *P*<.001; Cohen *d*=1.51) and on the delayed final exam (mean 80.96, SD 8.47 vs mean 78.62, SD 6.63; *P*=.02; Cohen *d*=0.31). Significant improvements were observed in information gathering, hypothesis generation, and differential diagnosis (all *P*<.001 after Holm correction), while treatment/management planning did not differ (*P*=.19). The intervention group reported higher perceived difficulty, perceived improvement in clinical reasoning, engagement, transfer of self-efficacy, and evaluation of the course materials (all *P*<.001). Voluntary responses (mean 2.30, SD 1.11 vs mean 1.68, SD 0.92; *P*=.008), evidence-referencing statements (mean 1.90, SD 0.88 vs mean 1.45, SD 0.71; *P*=.01), and net group discussion time (mean 36.50, SD 6.88 vs mean 28.42, SD 7.02 minutes; *P*<.001) were all higher in the intervention group. The group-by-major interaction for the final examination was not statistically significant (*P*=.41). Audit logs showed that all AI-generated case drafts required expert correction.

**Conclusions:**

A multicomponent GenAI-assisted, 4-act progressive-disclosure CBL package implemented with rigorous human-in-the-loop verification was associated with higher case-analysis performance, modestly higher delayed examination performance, and greater behavioral engagement in one nonrandomized cohort. Randomized and class-level multilevel designs with longer follow-up are needed to determine the durability and generalizability of these findings.

## Introduction

Case-based learning (CBL) has become a widely adopted pedagogical approach in higher medical education, valued for its capacity to bridge theoretical knowledge and authentic clinical practice and to cultivate clinical reasoning skills [1,2]. Within preventive medicine and occupational health curricula, occupational case discussion is a core instructional component that links classroom concepts to public health and clinical decision-making. Compared with case-based instruction in purely clinical disciplines, occupational health case teaching demands that learners simultaneously master clinical diagnostic competencies and develop capabilities in epidemiologic investigation and occupational or environmental exposure assessment. However, CBL in occupational health education is often delivered in a single-session, static format, in which comprehensive clinical and exposure-related information is disclosed upfront. Such synchronous information disclosure prematurely curtails the iterative reasoning cycle that characterizes authentic clinical practice, in which evidence is gathered progressively and hypotheses are repeatedly calibrated. When information is overly complete, learners may adopt an “omniscient” perspective and become susceptible to premature diagnostic closure. They may circumvent the deliberate practice of hypothesis generation, evidence seeking, and hypothesis testing and refinement, instead relying on keyword matching and knowledge recall to reach solutions [3]. This tendency to jump to conclusions reflects a limitation in analytical thinking and may, in turn, be reinforced by learners’ drive for immediate certainty, encouraging reliance on mental shortcuts and avoidance of careful, deliberate reasoning [4]. Consequently, clinical reasoning may remain at the level of superficial information retrieval rather than deeper analytic inference, with insufficient training in identifying and prioritizing key information amid uncertainty and confounding cues. Traditional pedagogical models reliant on rote retrieval fall short in developing the higher-order clinical reasoning required in complex medical environments, underscoring the need to reform case-based pedagogy to enhance learners’ critical thinking and reasoning skills.

Unfolding case studies (UCSs) use progressive disclosure to promote active reasoning under uncertainty, closely mirroring the incremental decision-making of real clinical practice [5]. Although widely adopted in nursing education, UCS application in clinical and preventive medicine remains limited [3]. Designing these dynamic cases—particularly segmenting information and managing missing data—is highly resource-intensive and demands significant clinical and pedagogical expertise from instructors [6]. To address these barriers, the present study aims to develop practical UCS models tailored to clinical and preventive medicine, seeking an optimal balance between instructional realism and operational efficiency.

Situated Learning Theory (SLT) emphasizes the construction of authentic or high-fidelity contexts to promote learners’ cognitive processing and competence development within situated practice [7]. Although SLT has been incorporated into CBL to improve learning outcomes, several practical constraints remain [8]. From a cognitive load perspective, embedding plausible distractors into a case forces learners to stop passively absorbing information and start actively filtering evidence. This productive struggle generates germane cognitive load, which is essential for building robust clinical schemas [9]. However, in practical educational settings, medical contexts are inherently complex and uncertain, and teachers’ scenario design may be constrained by experience and thus become overly directional [9]. To reduce organizational burden, scenarios may be simplified, preserving only the most salient cues, and standardized cases may not be readily adaptable to learners from different disciplinary backgrounds. Moreover, the design of high-quality “distractor cues” and the development of multiple scenario variants are time- and labor-intensive. Accordingly, how SLT can be effectively integrated into CBL while improving the fidelity of real-world simulation has become a key direction in contemporary educational reform [10].

In recent years, AI, particularly generative AI (GenAI), has advanced rapidly; large language models now demonstrate substantial capabilities in knowledge integration and natural language generation [11,12]. GenAI has been increasingly applied to medical education and has been used to support the development of CBL resources, such as knowledge restructuring, knowledge-graph generation, production of visual materials, and drafting of assessment items [13]. Often referred to as “Socratic AI,” this approach uses targeted questioning to help students build clinical hypotheses and evaluate evidence, rather than simply handing them answers [14]. By guiding learners to articulate their reasoning step-by-step, it promotes much deeper cognitive engagement. Although these applications may reduce learners’ cognitive load and improve memorization efficiency, their contribution to cultivating reasoning potential and higher-order cognitive training remains limited. Other studies have explored the use of GenAI for SLT-based scenario simulation [15]. As a scalable generative engine, GenAI can produce high-fidelity “cognitive friction” scenarios in bulk and dynamically introduce complex and personalized distractor cues aligned with instructional objectives, thereby shifting AI from a passive content producer toward an active facilitator of uncertainty and complexity. Furthermore, the cognitive friction introduced by GenAI acts as a “task-relevant resistance” that drives deeper learning [16]. Aligned with the concept of “desirable difficulties,” this friction increases short-term effort but significantly improves long-term retention and clinical transfer. This productive friction inherently forces learners to abandon heuristics and systematically navigate diagnostic uncertainty. Nevertheless, GenAI is subject to hallucination, producing outputs that appear plausible but are factually inaccurate or fabricated. In the medical domain, erroneous contextual information can mislead learners and undermine appropriate reasoning development. The primary operational challenge is integrating GenAI efficiently while maintaining rigorous guardrails for clinical accuracy and educational safety.

To address the limitations of existing case-based pedagogy, we propose a multicomponent GenAI-assisted progressive-disclosure CBL package, a scripted, 4-act CBL model aligned with the epistemic structure of occupational medicine. This approach uses an “act” structure to simulate complex clinical contexts while explicitly incorporating occupational and environmental exposure attribution into the scenario trajectory. Through progressive disclosure, clinical information is released in stages and supplemented with validated distractor cues to emulate real-world diagnostic resistance and to prompt repeated cycles of hypothesis generation and evidence verification. In addition, a human-in-the-loop (HITL) mechanism is implemented, combining prompt engineering with expert review to ensure rigorous quality control of AI-generated content and to mitigate hallucination risk. Using occupational lead poisoning as a paradigmatic module, our quasi-experimental findings suggest that the GenAI-assisted progressive-disclosure CBL was associated with improvements in learners’ clinical reasoning and classroom engagement and with higher scores on selected reasoning processes theoretically related to premature diagnostic closure, thereby providing empirical support for a safety-focused pathway for pedagogical innovation.

## Methods

### Study Design and Participant Recruitment

This quasi-experimental study adopted a nonrandomized posttest design and was reported in accordance with the Transparent Reporting of Evaluations with Nonrandomized Designs (TREND) statement. The study was conducted at Chongqing Medical University during the 2024-2025 academic year and was approved by the Ethics Committee of Chongqing Medical University on September 14, 2024 (approval number: 2024-143). The study was conducted in accordance with the Declaration of Helsinki. All participants were informed of the study purpose, the voluntary nature of participation, and the confidentiality procedures and provided written informed consent prior to enrollment.

Participants were undergraduate students enrolled in the compulsory “occupational lead poisoning” teaching module. A total of 224 students from 3 administrative classes (Class of 2022) were included: clinical medicine (n=110), psychiatry (n=56), and medical laboratory science (n=58). Because curricular allocation followed the administrative class structure, allocation was implemented at the class and teaching-session level rather than at the individual level. Students in each intact teaching session were assigned to the same study arm, resulting in an intervention group (n=114; clinical medicine 56, psychiatry 28, and medical laboratory science 30) and a control group (n=110; clinical medicine 54, psychiatry 28, and medical laboratory science 28). Because each public health laboratory classroom accommodated a maximum of approximately 36 students, the clinical medicine class was taught in 2 parallel teaching sessions and the psychiatry and medical laboratory science classes in one session each within each study arm, yielding 8 teaching sessions in total (4 per arm). After entering the classroom, students were randomly organized into small groups, with a maximum of 6 students per group, for classroom discussion and group tasks. Baseline equivalence between groups was assessed for age, sex distribution, and grade point average (GPA) in the prerequisite course. The inclusion criteria were as follows: (1) undergraduate students in clinical medicine, psychiatry, or medical laboratory science; (2) completion of prerequisite theoretical teaching and full attendance in the case-based teaching sessions; and (3) voluntary participation with written informed consent. The exclusion criteria were as follows: (1) an absence rate >20%; (2) failure to complete key outcome assessments; and (3) missing or clearly implausible data that were unsuitable for analysis.

Because the module was compulsory, all scheduled students were invited to participate. No participant was excluded, and all participants completed the intervention and outcome assessments. Thus, the analytic sample was identical to the recruited sample (intervention: n=114; control: n=110).

To ensure adequate statistical power and to mitigate selection bias commonly encountered in nonrandomized educational studies, an a priori power analysis was conducted using G*Power (version 3.1; Heinrich-Heine-Universität Düsseldorf). Assuming a 2-tailed independent-samples *t* test with an α of .05, 80% power, and a moderate effect size (Cohen *d*=0.5), the minimum required sample size was 128. The final sample of 224 exceeded this requirement, providing adequate power to detect moderate-to-large intervention effects on the primary outcomes. Because students were allocated by administrative class and teaching session, this individual-level calculation may overestimate the effective sample size if class-level clustering is substantial; clustering-aware sensitivity analyses are described in the Statistical Analysis section.

### Setting and Instructors

A total of 3 instructors with relevant teaching experience in the course and similar pedagogical backgrounds and experience in occupational medicine participated in the teaching sessions and taught across both study arms on a rotating basis. Before the teaching sessions, all instructors underwent standardized training and participated in collective lesson preparations to harmonize the learning objectives, core knowledge points, classroom tasks, and class duration. Apart from the specific components of the GenAI-assisted progressive-disclosure CBL package, including staged disclosure, structured reasoning tasks, and multimodal materials, the 2 groups received the same teaching topic, comparable class duration, and the same basic instructional requirements. We acknowledge that instructor-level differences and other unmeasured factors, such as prior exposure to CBL, digital literacy, learning motivation, and previous clinical-reasoning experience, may have influenced the results and are addressed as limitations.

### Teaching Intervention

Each case discussion session lasted 3 class hours (45 minutes per class hour). Both groups covered the same learning objectives, core content, and contact hours on occupational lead poisoning; only the case-delivery format differed between groups. The control group used a static case format, whereas the intervention group used a GenAI-assisted 4-act progressive-disclosure format while covering the same knowledge points.

#### Control Group

Students received conventional CBL, in which the full, static case record (including chief complaint, history, physical examination, laboratory findings, occupational exposure history, and workplace context) was disclosed at the beginning of the session. Classroom activities followed a linear workflow (“presentation → discussion → summary”), primarily emphasizing information extraction and diagnosis matching.

#### Intervention Group

Students received the GenAI-assisted progressive-disclosure CBL package [17,18], which used a scripted 4-act internal structure and progressively released key information together with teacher-verified distractor cues across acts.

The 4-act structure mapped onto the real-world workflow for recognizing and managing occupational poisoning and aligned with a staged progression of learners’ cognitive processes from cue detection to evidence weighting, causal attribution, and intervention decision-making. To prevent premature “answer-matching” once information became sufficient, each act provided only the minimum information required for the current task, while other cues were deliberately retained as “to be elicited/to be verified.” This approach prompted students to generate structured question lists, propose subsequent diagnostic tests or field investigations, and update their diagnostic hypotheses as new evidence became available.

With respect to scenario and competence design, Act 1 focused on problem framing and initial hypothesis generation under nonspecific symptoms. Act 2 emphasized occupational history elicitation and laboratory cues to train evidence identification, evidence grading, and diagnostic calibration. Act 3 shifted to field investigation and exposure attribution, training learners to integrate individual clinical information with workplace/environmental evidence to infer the “exposure–environment–health effect” causal chain. Act 4 addressed management and prevention, training learners to balance clinical treatment with occupational health interventions and to formulate action plans integrating individual therapy and population-level prevention, followed by reflective summarization and transfer to new contexts.

Across the acts, GenAI served as a “scenario driver” and “instructional scaffolding” tool. Specifically, GenAI was used to rapidly construct high-fidelity clinical encounter scenarios using multimodal materials (eg, images and audio) and to generate controllable nonspecific distractor cues in Act 1; to present structured dialogue scripts for occupational history probing and to externalize key evidence-threshold relationships through mind maps or knowledge graphs in Act 2; to provide visualizations and checklist-style prompts for workplace assessment and field investigation in Act 3; and to support synthesis of treatment and occupational health intervention points using knowledge graphs and decision frameworks in Act 4. Importantly, all GenAI-generated materials and cues were reviewed and validated by instructors before classroom deployment to ensure medical accuracy and instructional safety.

To minimize contamination bias (ie, cross-group sharing of case details), the 2 groups were scheduled in parallel time slots within the same week to reduce opportunities for information exchange. In addition, all students signed an academic integrity agreement not to disseminate the 4-act structure or distractor cues prior to study completion. During the intervention, GenAI was used solely on the instructor side for instructional material development. To assess students’ independent clinical reasoning, the use of GenAI tools by students was prohibited during in-class discussions and the final case-analysis assessment. After the class, students were asked to limit any subsequent discussions to their general learning experiences and perceptions of the course and not to discuss specific case content, implementation procedures, or instructional methods.

**Table 1:** Scripted “4-act” case-based learning design for the lead poisoning case.

Act	Scenario	Key cues and prompts	Generative AI support	Targeted competencies
Act 1	Illness onset and clinical encounter	Nonspecific symptoms/signs (eg, fatigue, arthralgia, right upper-limb numbness, and abdominal pain)	Rapid construction of the encounter scenario (multimodal visualization via text-to-image/audio); generation of controllable distractor cues (eg, pallor and constipation)	Problem framing and initial hypothesis generation
Act 2	Occupational history and laboratory cues	Battery-factory exposure history; elevated blood/urine lead levels; signs such as a “lead line”	Dialogue-based scenario for targeted occupational-history probing; mind maps/knowledge graphs (eg, blood/urine lead grading and diagnostic thresholds)	Evidence identification and grading
Act 3	Field investigation and exposure attribution	Insufficient engineering controls, inadequate PPE, airborne lead exceeding limits, etc.	Visualization of the workplace/operational environment; scenario-based field investigation; structured presentation of sampling/testing indicators and cues	Exposure–health effect causal attribution
Act 4	Management and prevention decision-making	Standardized treatment, occupational health interventions, and tertiary prevention	Knowledge graphs/mind maps to support synthesis of key points and transfer-oriented summarization	Integrated decision-making and reflective practice

### HITL Quality Control Protocol

To address the inherent risks of AI hallucinations, defined as the generation of plausible but clinically inaccurate or fabricated information, a formal HITL quality control protocol was established. Under this protocol, the GenAI tool was strictly confined to a “faculty-controlled development environment” (sandboxed environment). At no point were students granted direct, unmediated access to the raw model output.

All toxicological parameters or excerpts of real clinical records used to generate virtual patient data were deidentified in accordance with applicable privacy standards (eg, China's Personal Information Protection Law). In addition, the model’s data retention option was disabled to ensure that the proprietary educational case scripts were not stored or used for subsequent model training. The case-generation process followed a 3-stage verification cycle:

1. Iterative prompt engineering: faculty members used structured prompts to generate the initial 4-act script, clinical distractors, and virtual patient dossiers. Prompt templates are provided in
  Multimedia Appendix 1
  .
2. Expert blind review: a panel of 3 senior occupational medicine specialists (PS, CC, and SX), blinded to the AI’s initial drafts, reviewed all generated content for clinical accuracy, toxicological realism, and alignment with the National Medical Licensing Examination standards. The panel comprised occupational medicine faculty members, each with more than 3 years of teaching experience, who also performed the hallucination filtering and correction stage and recorded the audit metrics.
3. Hallucination filtering: instances of “hallucinated” data—such as incorrect laboratory reference ranges or illogical environmental exposure histories—were manually corrected or regenerated. The number of errors, normative edits, and review time per draft were recorded to enable transparency reporting in accordance with the GenAI in Medical Research (GAMER) statement.

This HITL framework intended to support the use of GenAI as an efficient pedagogical engine while remaining under strict human oversight, thereby safeguarding the educational and ethical integrity of the intervention.

### GenAI Implementation and Prompt Engineering

#### Tools and Prompting

DeepSeek-V3 was used as the underlying model to generate multimodal case materials, patient personas, and stage-specific distractor information. The prompting template included explicit role specification (eg, “act as an expert in occupational toxicology”), clinical constraints (eg, “blood lead levels must comply with Chinese national diagnostic criteria for occupational chronic lead poisoning, GBZ 37-2015”), and a structured output format (see Multimedia Appendix 1). In addition, this study followed the GAMER reporting guidelines [19]. We explicitly detail the model specifications, prompt histories, human oversight workflows, and privacy measures. The completed GAMER checklist is provided in Multimedia Appendix 2.

#### Content Verification and Quality Control

To mitigate inaccuracies in AI-generated outputs, a standard operating procedure (SOP) was established [19]. Faculty members reviewed the generated materials for medical thresholds, logical consistency, terminology, and alignment with occupational health teaching objectives before classroom use. All AI drafts were independently reviewed by 3 senior instructors in occupational medicine (the same expert panel described in “HITL Quality Control Protocol”) against current national occupational health standards and clinical guidelines. Critical thresholds (eg, lethal doses and absolute contraindications) were manually checked before any material was approved for classroom use.

#### Student-Use Policy

During the intervention, GenAI was used solely on the instructor side for instructional material development. To assess students’ independent clinical reasoning, the use of GenAI tools by students was prohibited during in-class discussions and the final case-analysis assessment.

### Outcome Measures

The outcome measures covered both learning outcomes and learning processes, including objective academic performance, subjective learning experience, and objective behavioral indicators of classroom engagement. To manage the multiplicity of statistical tests, a hierarchical analytic plan was defined. The case-analysis assignment score was the sole primary outcome; the delayed final examination score, the 4 reasoning-dimension scores, and the 5 learning experience domain ratings were secondary outcomes; and the 3 behavioral indicators and the subgroup analyses by major were exploratory process and generalizability outcomes.

#### Primary Outcome: Standardized Case-Analysis Assignment Score (0-100)

This measure was used to evaluate students’ knowledge integration and knowledge-transfer performance in an authentic context. Each student completed the assignment independently. The assignment was uniformly designed and deidentified and was scored independently by 2 raters who were blinded to group allocation; interrater reliability was assessed using the intraclass correlation coefficient (ICC). To reduce subjectivity in scoring open-ended responses, an analytic rubric was used to standardize grading. Each question was decomposed into observable key scoring points, with partial-credit and deduction rules predefined. Prior to formal scoring, rater training and calibration were conducted, and a rescoring/adjudication rule was established (eg, third-party review was initiated when the discrepancy between the 2 raters exceeded a predefined threshold). In addition, the total score was further decomposed into 4 clinical reasoning dimensions (25 points each; total 100 points), corresponding to information gathering, hypothesis generation, differential diagnosis, and treatment/management planning, to examine the differential effects of the intervention across reasoning components. The case-analysis assignment used a new clinical case with novel contextual details and was not identical to the case used in the teaching session; the rubric was developed by the teaching team before the intervention on the basis of the 4 target reasoning dimensions, and students did not have access to the scoring rubric during teaching. Interrater reliability for the case-analysis scoring was excellent (ICC=0.91).

#### Secondary Outcome: Final Examination Score (0-100)

The final examination was administered 3-4 weeks after completion of the case-based teaching module and served as a delayed posttest to assess short-term retention and transfer of the intervention effects several weeks later. The examination was a closed-book test with a total score of 100 points, consisting of 40 points for subjective items and 60 points for objective items. The subjective section included term explanations, short-answer questions, and case-analysis questions, whereas the objective section covered knowledge points from the preventive medicine curriculum. Within the examination, case analysis–related items accounted for 15 points, lead poisoning–related objective items accounted for 5 points, and reasoning-oriented objective items accounted for approximately 10 points. Students in both groups took the same course examination using versions A and B; the 2 versions contained the same item content but used different option orders to reduce potential examination-related interference. Item writing and grading were conducted while blinded to students’ group allocation. The final examination scores were independently scored by 2 raters blinded to group allocation, with excellent interrater reliability (ICC=0.92). The reliability of the examination was verified according to the routine quality-control procedures for the course examination. Because of institutional examination-confidentiality requirements, the complete examination paper cannot be publicly released, and item-level analyses by question type could not be further conducted.

#### Secondary Outcome: Subjective Learning Experience

Students completed a structured self-report questionnaire using a 0-10 point scale to rate five dimensions: (1) perceived difficulty, (2) perceived improvement in clinical reasoning, (3) engagement, (4) self-efficacy (confidence in transferring the reasoning framework to new contexts), and (5) evaluation of the course materials, which included the GenAI-generated elements in the intervention group. Details of the instrument are provided in Multimedia Appendix 3. Each domain comprised 4 items (Multimedia Appendix 3), and the domain score was the mean of its 4 items. Internal consistency was assessed with Cronbach α within each domain (α=0.77-0.91); because the 5 domains measure conceptually distinct constructs, an overall coefficient across domains was not interpreted as evidence of scale reliability, and each domain was analyzed as its own rating. The ratings were exploratory and should not be interpreted as objective evidence of educational effectiveness.

#### Exploratory Process Outcomes: Objective Behavioral Indicators of Classroom Engagement

To objectively validate students’ self-reported learning engagement and further capture behavioral changes during the classroom learning process, video recordings of the sessions were reviewed and analyzed. A behavioral test was performed by trained instructors using a standardized question list developed during collective lesson preparation (10 questions designed for individual responses and 6 questions designed for group discussion followed by representative responses). During the sessions, the instructor used a random-calling strategy to select individual students and representative groups to answer these structured questions. Consequently, a total of 80 individual responses (10 questions×8 teaching sessions; 40 per arm) and 48 group responses (6 questions×8 teaching sessions; 24 per arm) were captured in the video recordings and fully coded for behavioral metrics. Because of constraints related to course scheduling and teaching implementation, repeated measurements of these classroom behavioral indicators were not conducted. In addition, voluntary responses may have been influenced by the specific questions posed. Therefore, these indicators were reported descriptively as exploratory process measures and were used to support the interpretation of changes in student engagement behaviors.

Three classroom behavioral indicators were extracted during the case discussions: (1) the number of voluntary responses to questions, counted at the individual student level, (2) the number of evidence- or reference-based statements, counted at the individual student level, and (3) net group discussion duration, recorded at the group level in minutes per group. These indicators were used to assess, from a behavioral perspective, whether the teaching model promoted a shift in students’ learning behaviors from passive reception to active inquiry.

A “voluntary response” was defined as a response initiated by a student after the teacher posed a question, without being called on or required to answer by the teacher. This included voluntarily raising a hand to answer or actively communicating with the teacher regarding the question. Responses resulting from teacher nomination, direct assignment, or mandatory questioning were not counted as voluntary responses; “The number of evidence- or reference-based statements” is defined as the number of times a student, when answering a question, cites in-class clinical materials or diagnostic standards to support their answer. “Net group discussion duration” was defined as the period during which more than 2 students within a group interacted with each other in the video recording; the discussion was considered to have ended when no student interaction was observed.

#### Exploratory Outcome: Subgroup Comparisons

To evaluate the applicability of the intervention across educational backgrounds, subgroup comparisons of final exam scores were conducted by major (clinical medicine, medical laboratory science, and psychiatry), together with group-by-major interaction tests, as exploratory outcomes.

### AI-Generated Occupational Exposure Case Development and Expert Audit

To evaluate and control the quality of AI-generated occupational medicine cases, we developed a structured HITL audit protocol. The teaching content covered representative occupational hazards commonly included in undergraduate occupational medicine training, including lead poisoning, arsenic exposure, hydrogen sulfide poisoning, and dust-related occupational lung disease.

For each teaching topic, a hazard-specific key prompt was constructed to guide the large language model in generating an occupational exposure case. The prompt required the model to produce a staged clinical scenario involving occupational history, exposure pathway, clinical manifestations, laboratory or environmental monitoring findings, differential diagnosis, and prevention or management measures. The model was instructed to align case details with relevant national occupational health standards and diagnostic criteria and to include plausible distractors that could support progressive disclosure and clinical reasoning training.

All AI-generated case drafts were subsequently reviewed by 3 occupational medicine faculty members, each with more than 3 years of teaching experience. The reviewers independently assessed each case according to national occupational disease diagnostic standards, occupational exposure limit criteria, and routine teaching requirements. During the review process, each teacher recorded:

1. The number of medical indicator or threshold errors identified and corrected
2. The number of logical inconsistencies or unreasonable plots identified and rectified
3. The number of terminology or expression revisions made for standardization
4. The total review and correction time in minutes

Medical indicator or threshold errors included incorrect diagnostic thresholds, inappropriate laboratory reference values, unreasonable exposure concentrations, or inconsistency with national diagnostic standards. Logical inconsistencies referred to contradictions between exposure history, clinical manifestations, laboratory findings, and disease progression. Terminology and expression revisions referred to modifications of nonstandard occupational health terminology, ambiguous descriptions, or wording inconsistent with teaching norms.

The final quality-control indicators were calculated at the case level. A total of 25 case drafts were generated (lead poisoning n=6, arsenic n=6, hydrogen sulfide n=7, and dust-related occupational lung disease n=6), and each draft was independently reviewed by 3 instructors, yielding 75 reviewer assessments. For each case, the mean number of errors or revisions identified by the 3 reviewers was calculated; descriptive statistics were then reported as mean (SD) across the 25 cases.

### Statistical Analysis

Statistical analyses were conducted using R (version 4.5.1; R Core Team) software. Continuous variables are reported as mean (SD), and categorical variables as frequencies and percentages. Baseline characteristics were compared between groups using independent-samples Student *t* tests for continuous variables and chi-square tests for categorical variables; standardized mean differences (SMDs) were reported for continuous baseline variables. Effect sizes for continuous outcomes were quantified using Cohen *d* (with conventional thresholds: small ≥0.2, medium ≥0.5, large ≥0.8), and 95% CIs for *d* were computed from the noncentral *t* distribution. For between-group comparisons of the primary case-analysis score and the final examination score, we used independent-samples *t* tests. To evaluate the robustness of the main comparisons to baseline imbalance, analysis of covariance (ANCOVA) models adjusted for prerequisite-course GPA were fitted for both outcomes. The 4 clinical reasoning dimension scores, the 5 subjective-experience domains, and the 3 behavioral indicators were treated as secondary or exploratory outcomes, with the Holm-Bonferroni correction applied within each outcome family to control the family-wise type I error rate. The 3 subgroup comparisons by major were treated as exploratory; the formal test for differential treatment effects across majors was a group-by-major interaction term in a 2-way ANOVA, rather than the statistical significance of individual subgroup tests.

Interrater reliability for both case-analysis scoring and final examination scoring was assessed using a 2-way random-effects ICC with absolute agreement. Behavioral discussion-duration data were analyzed at the group level, consistent with their unit of measurement; voluntary responses and evidence-referencing statements were analyzed at the individual-student level. Missing data were handled by complete-case analysis because the rate of missingness was negligible (<1%). All tests were 2-tailed, and *P*<.05 was considered statistically significant unless otherwise specified.

Because allocation was implemented at the class and teaching-session level, students within a session cannot be regarded as fully independent observations. As a sensitivity analysis, we estimated ICCs using academic major as a proxy clustering variable; the estimated ICCs were approximately 0 for both objective outcomes. We also computed cluster-robust standard errors at the 6 group-by-major strata (3 majors×2 study arms), which corroborated the primary results. Administrative-class identifiers were not retained in the deidentified dataset, so a formal class-level multilevel model could not be fitted; this is acknowledged in the Limitations.

## Results

### Participant Flow and Baseline Equivalence

A total of 224 undergraduate students completed the study (intervention group, n=114; control group, n=110). No participants were excluded or withdrew, and no primary outcome data were missing; the analytic sample was identical to the enrolled sample (Figure 1). Baseline characteristics were compared prior to outcome comparisons. As shown in Table 2, there were no statistically significant between-group differences in age, sex distribution, or academic performance in the prerequisite occupational medicine course (all *P*>.05). These results suggest similar measured baseline characteristics on the measured variables; however, nonsignificant baseline comparisons do not establish equivalence, and unmeasured characteristics (eg, prior CBL exposure and motivation) may still differ between groups.

**Figure 1:**
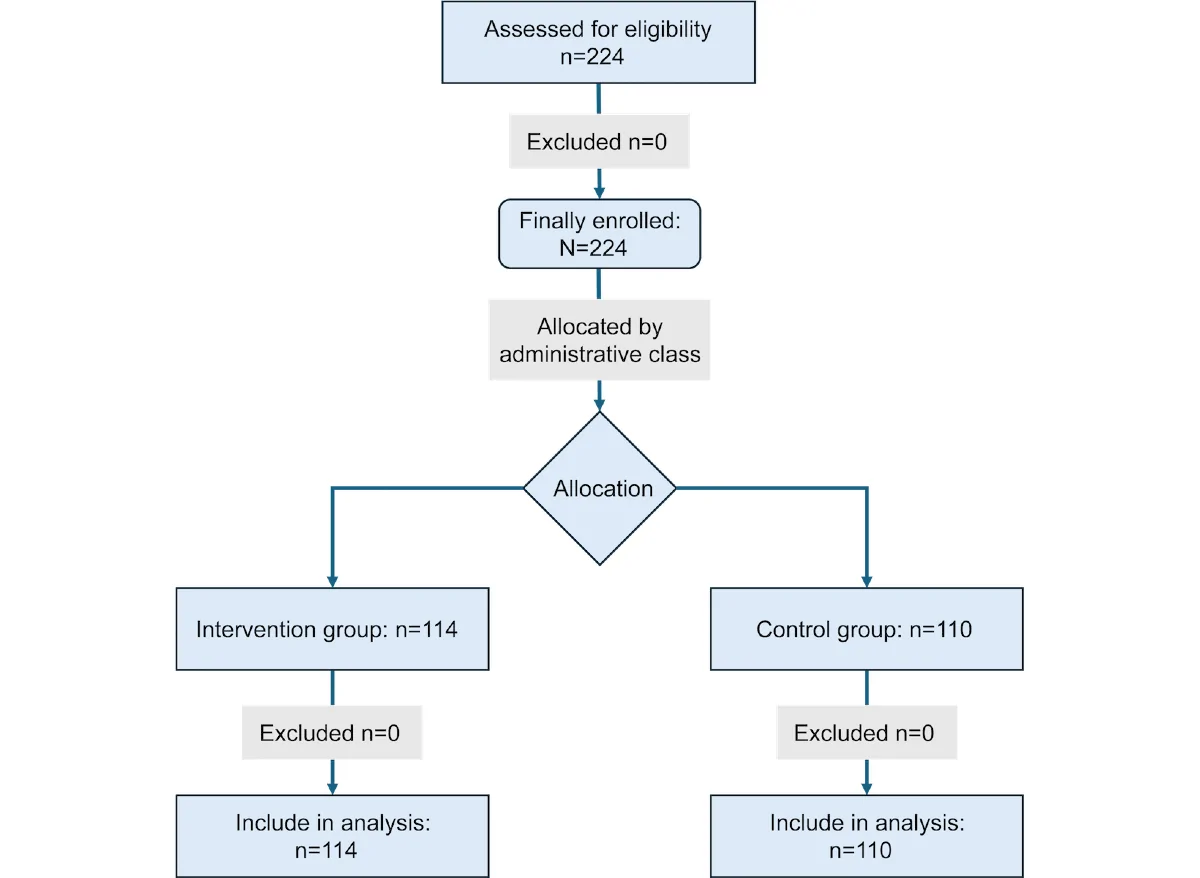
Flowchart of participation.

**Table 2:** Baseline characteristics of the participants

Characteristic	Intervention group (n=114)	Control group (n=110)	Test statistic	*P* value	Standardized difference
Age (years), mean (SD)	20.4 (1.2)	20.6 (1.1)	–1.33 (222)^a^	.20	–0.17^b^
Sex (male/female)	54/60	50/60	0.10 (1)^c^	.75	0.02^d^
Prior toxicology GPA^e^, mean (SD)	78.41 (4.65)	78.55 (8.24)	–0.15 (222)^a^	.88	–0.02

^a^*t* test (*df*) value.

^b^Standardized mean difference.

^c^Chi-square (*df*) values.

^d^Cramér V.

^e^GPA: grade point average.

### Objective Learning Outcomes

Objective learning outcomes were assessed using the postclass case-analysis score and the final examination score. Interrater reliability was good for the case-analysis grading (ICC=0.91) and for the final examination scoring (ICC=0.92). For the case analysis, the intervention group achieved significantly higher scores than the control group (mean 88.41, SD 3.85 vs mean 82.51, SD 3.94; *P*<.001), with a large effect size (Cohen *d*=1.51, 95% CI 1.23-1.83). Next, the final examination score was also compared, showing higher performance in the intervention group (mean 80.96, SD 8.47 vs mean 78.62, SD 6.63; *P*=.02), with a small-to-moderate effect size (Cohen *d*=0.31, 95% CI 0.04-0.57). The divergence between the case-analysis effect (Cohen *d*=1.51) and the final examination effect (Cohen *d*=0.31) suggests that the intervention most strongly reinforced the structured reasoning workflow practiced in the case-analysis task, while effects on broader knowledge consolidation were more modest. Cluster-robust sensitivity analyses at the 6 group-by-major strata corroborated these conclusions. Details are presented in Table 3.

**Table 3:** Comparison of objective learning outcomes between the 2 groups.

Outcome measure	Intervention group (n=114), mean (SD)	Control group (n=110), mean (SD)	Mean difference (95% CI)	*P* value	Cohen *d* (95% CI)
Case analysis score (0-100)	88.41 (3.85)	82.51 (3.94)	5.90 (4.87-6.93)	<.001	1.51 (1.23-1.83)
Final exam score (0-100)	80.96 (8.47)	78.62 (6.63)	2.33 (0.33-4.33)	.02	0.31 (0.04-0.57)

### Subdimensional Clinical Reasoning Performance

To clarify the impact of the GenAI-assisted progressive-disclosure CBL package on domain-specific reasoning competencies, the total case-analysis score was decomposed into 4 core dimensions (25 points each). As shown in Table 4, the intervention group outperformed the control group in information gathering (mean 22.66, SD 1.87 vs mean 20.46, SD 2.13), hypothesis generation (mean 22.28, SD 1.99 vs mean 20.47, SD 2.06), and differential diagnosis (mean 22.34, SD 1.96 vs mean 20.72, SD 2.09), with all differences reaching statistical significance (all *P*<.001) and corresponding to large effect sizes (Cohen d≈1.10, 0.89, and 0.80, respectively). By contrast, the difference in treatment and management planning did not reach statistical significance (mean 21.13, SD 1.56 vs mean 20.86, SD 1.55; *P*=.19; Cohen *d*=0.18).

**Table 4:** Subdimensional scores for clinical reasoning in the case analysis worksheet.

Reasoning dimension (maximum 25 points each)	Intervention group (n=114), mean (SD)	Control group (n=110), mean (SD)	Mean difference (95% CI)	*P* value	Cohen *d*	Holm-adjusted *P* value
Information gathering	22.66 (1.87)	20.46 (2.13)	2.20 (1.67 to 2.73)	<.001	1.10	<.001
Hypothesis generation	22.28 (1.99)	20.47 (2.06)	1.80 (1.27 to 2.34)	<.001	0.89	<.001
Differential diagnosis	22.34 (1.96)	20.72 (2.09)	1.62 (1.09 to 2.16)	<.001	0.80	<.001
Treatment and management plan	21.13 (1.56)	20.86 (1.55)	0.27 (–0.14 to 0.68)	.19	0.18	.19

This pattern is consistent with the conceptual framework underlying the progressive-disclosure design. The progressive disclosure and verified distractor cues may be particularly effective for strengthening hypothesis-evidence calibration during exploratory reasoning, whereas treatment and management planning relies more heavily on retrieval and application of standardized guideline-based knowledge, which can also be adequately supported by conventional CBL. We interpret this differential pattern as consistent with—rather than direct evidence of—the hypothesized mechanism, rather than as a generic “more is better” effect on all reasoning dimensions.

### Subgroup Analysis by Major

To explore whether the intervention effect varied across educational backgrounds of the GenAI-assisted progressive-disclosure CBL package across educational backgrounds, we conducted subgroup analyses by major and compared final examination scores (Table 5). The intervention group scored higher than the control group in all 3 majors; however, statistical significance was observed only among students in clinical medicine (mean 81.55, SD 8.63 vs mean 78.66, SD 6.27; *P*=.05). Differences were not statistically significant in psychiatry (mean 81.05, SD 8.22 vs mean 77.50, SD 6.39; *P*=.08) or medical laboratory science (mean 79.75, SD 8.55 vs mean 79.68, SD 7.57; *P*=.97). After Holm-Bonferroni adjustment across the 3 subgroup comparisons, none of the differences remained statistically significant (adjusted *P*=.14, .15, and .97, respectively). The group-by-major interaction for the final examination was not statistically significant (*F*_2,218_=0.88; *P*=.41), indicating no reliable evidence that the intervention effect differed by major. Scores did not differ significantly across majors overall (*F*_2,221_=0.23; *P*=.79). Overall, these exploratory findings suggest that the intervention effect was directionally larger in clinically oriented disciplines, but the limited subgroup sample sizes and the nonsignificant interaction test warrant cautious interpretation and validation in larger, multicenter studies.

**Table 5:** Subgroup analysis of final examination scores by academic major.

Academic major	Intervention group score	Control group score	Mean difference (95% CI)	Exploratory *P* value
Clinical medicine	81.55 (8.63); (n=56)	78.66 (6.27); (n=54)	2.90 (0.00 to 5.79)	.05
Psychiatry	81.05 (8.22); (n=28)	77.50 (6.39); (n=28)	3.55 (–0.42 to 7.53)	.08
Laboratory medicine	79.75 (8.55); (n=30)	79.68 (7.57); (n=28)	0.07 (–4.12 to 4.35)	.97

### Subjective Learning Experience

To control the family-wise type I error arising from multiple comparisons among the 5 domains, a Holm-Bonferroni correction was applied. Internal consistency was acceptable to high across the 5 domains (Cronbach α: perceived difficulty 0.77; reasoning improvement 0.85; engagement 0.83; self-efficacy 0.84; course materials 0.91). As shown in Table 6, the intervention group reported significantly higher scores for perceived improvement in clinical reasoning (mean 8.55, SD 0.50 vs mean 5.96, SD 0.79; *P*<.001) and course engagement (mean 8.46, SD 0.50 vs mean 6.11, SD 0.81; *P*<.001) than the control group. Self-efficacy for transferring the reasoning framework to new contexts was also higher in the intervention group (mean 8.58, SD 0.50 vs mean 6.13, SD 0.80; *P*<.001). Meanwhile, the intervention group perceived greater case difficulty (mean 7.53, SD 0.50 vs mean 5.98, SD 0.80; *P*<.001), suggesting that progressive disclosure and teacher-verified AI distractor cues increased cognitive challenge and demanded more rigorous hypothesis testing, consistent with the desirable-difficulty framing introduced in the Introduction. In addition, ratings for evaluation of course materials were substantially higher in the intervention group than in the control group (mean 8.54, SD 0.88 vs mean 5.44, SD 0.93; *P*<.001), suggesting a more positive evaluation of the course materials. All 5 comparisons remained statistically significant after the Holm-Bonferroni adjustment (adjusted *P*<.001).

**Table 6:** Student subjective evaluation of the learning experience (scale: 0-10).

Questionnaire domain	Intervention group (n=114)	Control group (n=110)	Mean difference (95% CI)	Unadjusted *P* value	Holm-adjusted *P* value
Perceived difficulty	7.53 (0.50)	5.98 (0.80)	1.54 (1.37-1.72)	<.001	<.001
Clinical reasoning enhancement	8.55 (0.50)	5.96 (0.79)	2.60 (2.42-2.77)	<.001	<.001
Course engagement	8.46 (0.50)	6.11 (0.81)	2.36 (2.18-2.53)	<.001	<.001
Self-efficacy	8.58 (0.50)	6.13 (0.80)	2.45 (2.28-2.63)	<.001	<.001
Evaluation of course materials	8.54 (0.88)	5.44 (0.93)	3.10 (2.86-3.34)	<.001	<.001

### Objective Classroom Engagement and Behavioral Metrics

The self-reported increase in engagement was supported by objective behavioral indicators derived from classroom video analysis (Table 7). Compared with the control group, students in the intervention group responded voluntarily to questions more frequently (mean 2.30, SD 1.11 vs mean 1.68, SD 0.92; *P*=.008). The intervention group also produced significantly more evidence- or reference-based statements (mean 1.90, SD 0.88 vs mean 1.45, SD 0.71; *P*=.01). The progressive-disclosure structure also significantly increased net group discussion time (mean 36.50, SD 6.88 vs mean 28.42, SD 7.02 minutes; *P*<.001), suggesting more intensive interaction and deeper discussion. All 3 behavioral differences remained significant after the Holm-Bonferroni adjustment across the 3 indicators (adjusted *P*=.02, .02, and <.001, respectively). Although the frequency of evidence-referencing statements was higher in the intervention group, the absolute frequencies remained low in both groups, and the formal quality of the cited evidence was not assessed; this remains an area for further instructional development.

**Table 7:** Objective behavioral metrics of student engagement during case discussions.

Behavioral metric	Intervention group (n=114), mean (SD)	Control group (n=110), mean (SD)	*P* value
Voluntary responses	2.30 (1.11)	1.68 (0.92)	.008
Information/reference search frequency	1.90 (0.88)	1.45 (0.71)	.01
Net group discussion duration (minutes)	36.50 (6.88)	28.42 (7.02)	<.001

### AI Hallucination Mitigation and Quality Control Metrics

In accordance with the GAMER statement on transparency in reporting AI use, we quantified the HITL quality-control process during case development. Across the generated case drafts (each independently reviewed by 3 occupational medicine faculty members; per-case values are the mean of the 3 reviewers), every draft required expert correction. Independent instructor audit logs showed that each AI-generated case contained, on average, 1.65 (SD 0.68) errors in medical indicators/thresholds and 2.43 (SD 1.08) instances of logical inconsistency. To standardize terminology and expression, instructors implemented an average of 3.12 (SD 1.12) normative edits per case. The mean time required for expert review and correction of one AI-generated draft was 18.72 (SD 2.89) minutes. These findings indicate that, prior to classroom deployment, domain-expert oversight remains indispensable for mitigating hallucination risk and ensuring instructional safety (Table 8).

**Table 8:** Quantification of the human-in-the-loop audit for AI-generated cases.

Quality control metric	Occurrences/time per case, mean (SD)
Medical indicators/threshold errors corrected	1.65 (0.68)
Logical inconsistencies/unreasonable plots rectified	2.43 (1.08)
Terminology/expression normative revisions	3.12 (1.12)
Average expert review and audit time (minutes)	18.72 (2.89)

## Discussion

### Principal Findings

Our findings indicate that the GenAI-assisted progressive-disclosure CBL package was associated with significantly higher clinical reasoning performance among learners, higher scores on reasoning dimensions theoretically related to premature closure, and, to some extent, greater willingness to engage in active classroom participation. The observed benefits also suggest a degree of cross-disciplinary applicability, although the nonsignificant group-by-major interaction indicates that differential effects across majors were not established. It should be emphasized that the proposed 4-act structure is not an entirely novel design; rather, it is conceptually grounded in the well-established tradition of UCS [5,20]. The core pedagogical mechanism of UCS lies in progressive disclosure, whereby information is released in stages so that learners repeatedly cycle through hypothesis generation, information seeking, and judgment recalibration under uncertainty—more closely approximating how evidence emerges in real clinical care and occupational health practice. A recent scoping review further suggests that UCS-based designs are associated with overall positive performance outcomes in nursing education, including improvements in critical thinking, knowledge acquisition, and decision-making performance in situated tasks [5,21]. Building on this foundation, our 4-act design further distills key reasoning steps from authentic practice and places greater emphasis on contextual dynamics and the process nature of reasoning. An increase in student-initiated questions better reflects a learner’s active drive to seek information and test hypotheses under uncertainty, making it a highly process-sensitive measure of engagement. Furthermore, while the intervention yielded a large effect on case analysis scores (Cohen *d*=.51), this likely indicates improved “near transfer” of the specific reasoning workflow. Conversely, the smaller effect on the final examination (Cohen *d*=0.31) suggests only a modest gain in “far transfer”—the ability to integrate these skills into a broader knowledge base. The convergence between our results and the UCS literature provides additional support for the theoretical plausibility of progressive-disclosure CBL, while also indicating potential generalizability beyond a single discipline.

Regarding case construction, the “Scripted Remedies” approach proposed by Simms and Fox illustrates how GenAI can be leveraged to align narrative elements (eg, popular-culture motifs) with instructional goals to produce scripted cases with controllable complexity and narrative tension [22]. This is consistent with our design logic—namely, using AI to support efficient generation of scenarios and cues while maintaining instructor verification to safeguard accuracy. Consistent with the hypothesized mechanism, our dimension-level outcome analyses suggest that the intervention advantage was more pronounced in information gathering, hypothesis generation, and differential diagnosis, whereas differences in treatment and management planning were not statistically significant. This pattern implies that the scripted 4-act structure and verified distractor cues may preferentially strengthen exploratory reasoning under uncertainty, while treatment planning may rely more heavily on retrieval and application of established knowledge and guidelines—skills that can also be adequately supported by conventional teaching approaches. This suggests the intervention’s scope is bounded by exploratory reasoning; it should not be regarded as a general clinical-reasoning enhancer. Process indicators likewise showed that learners in the intervention condition demonstrated higher levels of engagement, including a significantly higher frequency of explicit evidence-referencing statements, which had previously been regarded as a nonsignificant trend in an earlier analysis; however, the absolute frequencies remained low in both groups, and the quality of the cited evidence was not assessed, so the formal quality and appropriateness of evidence-based argumentation remain areas for further instructional development. In subgroup analyses stratified by major, the small sample sizes in the psychiatry and medical laboratory science groups limited the statistical power to detect moderate between-group differences, and the group-by-major interaction was not statistically significant. Future studies with larger cohorts are needed to robustly confirm the intervention’s efficacy across these disciplines.

SLT has been widely applied in medical education [23]. Using Goffman’s dramaturgical metaphor, Cantillon et al [24] described how professional roles and norms are co-constructed through everyday interactions, informal learning, and collaborative work within clinical teams. From this perspective, clinical reasoning is rarely a purely linear “paper-and-pencil” logic exercise; rather, it resembles an evolving “performance” and “detective work” that requires iterative cue tracking and judgment recalibration under incomplete information, distributed responsibilities, and contextual pressures. Accordingly, the GenAI-assisted “4-act” scenario in the present study may be interpreted as a classroom-based micro-model of clinical ecology: by combining staged disclosure with role-based tasks, it allows learners to rehearse professional participation in a safer instructional environment, which is closely aligned with SLT’s emphasis on context-embedded learning. Related work on “Socratic AI” and adaptive tutoring for clinical CBL further proposes decomposing lengthy case narratives into discrete information nodes, releasing key cues only when learners ask the appropriate questions and complete necessary reasoning steps. This aligns with our “thresholded information disclosure” strategy and suggests that our intervention can be situated within an emerging family of adaptive, reasoning-oriented tutoring paradigms. In addition, questionnaire results indicated that learners perceived higher difficulty under the progressive-disclosure format, while simultaneously reporting higher engagement and self-efficacy. This pattern is consistent with Bjork’s concepts of desirable difficulties and productive struggle: moderate cognitive friction may not constrain learners but may instead motivate continuous hypothesis calibration and evidence-chain reconstruction, thereby facilitating deeper processing, a stronger sense of mastery, and greater learning motivation.

The application space of GenAI in medical education continues to expand. Potter and Jefferies [25] emphasized that GenAI may move beyond static scripts by enabling more dynamic, natural-language interaction with “living” virtual patients, potentially providing higher-fidelity contexts for communication and clinical reasoning training. In the present study, both process and outcome data suggest that the GenAI-assisted progressive-disclosure design can improve performance and promote engagement; the higher ratings for the course materials (which included GenAI-generated elements in the intervention group) also indicate an overall positive attitude toward integrating GenAI into the course [21,26]. Importantly, the value of GenAI in this study lies in its role as an efficient drafting tool whose outputs still required substantial expert verification; the available data do not allow us to claim reductions in total case-development time or cost, because no comparator based on conventional instructor-developed cases was included. Silvestri-Elmore and Burton [20] reported that AI and educational technologies can substantially alleviate the time and workload barriers that faculty face when designing complex case scripts (including UCS). Consistent with that view, the expert audit documented that review and correction of AI-generated drafts were required before classroom use; quantifying the net time saved relative to conventional case development is a task for future comparative studies.

Nevertheless, the capability boundaries and safety risks of GenAI in clinical reasoning should not be underestimated [27]. Although some models have been adapted using medical corpora and clinical knowledge [28,29], their reliability and safety remain contested [30]. Rao et al [31] reported that large language models may achieve high final-diagnosis accuracy when provided with complete case information (>90%), yet often perform poorly in early-stage differential diagnosis and reasoning navigation under information scarcity. This discontinuity has direct methodological implications for our instructional strategy: precisely because AI may be intrinsically less reliable in open-ended, information-incomplete reasoning phases, we deliberately avoided allowing learners to engage in unconstrained free-form dialogue with the model [32]. Instead, we adopted an expert-controlled, prescripted 4-act structure together with a human HITL verification and correction process. Consistent with this rationale, our audit data showed that AI-generated materials still required human correction of medical thresholds/values and logical inconsistencies, indicating that the unreviewed model outputs generated in this study were suitable for direct classroom deployment [33,34]. Accordingly, rather than advocating “AI replacing instructors,” our findings support a sustainable trajectory of human-AI collaboration, in which AI serves as a high-efficiency generator and scaffolding tool [35], whereas medical fact-checking, logical consistency review, and ethical/value judgments remain firmly human-led—particularly in domains such as clinical medicine, occupational medicine, and public health that depend heavily on standards, field evidence, and practice-based expertise. Our HITL workflow may therefore be viewed not only as an experimental control but also as a potentially transferable SOP for responsible integration of GenAI in educational settings. It should also be acknowledged that such a pathway increases demands on instructors. Educators require sufficient knowledge and practical experience to identify and correct AI errors in a timely manner.

This study has several limitations: (1) group assignment was based on administrative classes rather than individual-level randomization. Although baseline characteristics were comparable (SMD <0.2 for the measured variables), unobserved class-level factors could have influenced effect estimates, and the absence of class identifiers prevented a formal class-level multilevel analysis. (2) The intervention was a multicomponent package; progressive disclosure, the 4-act structure, distractor cues, multimodal materials, and differences in instructor facilitation were introduced together, so the independent contribution of GenAI cannot be isolated. (3) No preintervention baseline measure of clinical reasoning or the primary outcome was available; baseline similarity in demographic variables does not establish equivalence in reasoning ability, and unmeasured confounders (prior CBL exposure, motivation, digital literacy, and previous reasoning experience) cannot be excluded. (4) Teacher-level variables were not systematically measured or controlled. Delivering the GenAI-assisted progressive-disclosure CBL package typically requires stronger in-the-moment facilitation skills, including adaptive questioning, guidance under uncertainty, and fine-grained management of pacing and small-group interaction; implementation fidelity was not assessed with an independent quantitative rating instrument, and instructor enthusiasm and interaction style were not rated. (5) Students were not blinded to condition; favorable subjective ratings and behavioral engagement may partly reflect novelty effects, expectations, and greater instructor attention rather than pedagogical effectiveness per se. (6) Although parallel scheduling and academic-integrity agreements were used to reduce information leakage, cross-group contamination across groups could have occurred within the same school and peer networks, and no formal post hoc contamination checks (eg, social media monitoring or contamination-survey items) were conducted; this could attenuate between-group differences or introduce bias of uncertain direction. (7) As a single-center study, external validity is limited; multicenter replication is needed. (8) We assessed primarily short-term outcomes at module completion and 3-4 weeks thereafter; longer follow-up (eg, 3-6 months) is needed to determine the durability of intervention effects. (9) The case-analysis outcome was aligned with the taught reasoning workflow, so the large effect likely reflects near transfer; the smaller effect on the less-aligned final examination (Cohen *d*=0.31) suggests that far transfer remains uncertain. (10) Behavioral indicators were exploratory: they were coded by a single nonblinded coder without formal inter-rater reliability assessment, were available for a subsample of students and groups, and repeated observations within classes were not modeled. (11) Every audited GenAI draft required expert correction, and model specifications were only partially recorded, indicating that current GenAI outputs are not suitable for unsupervised classroom deployment and that reporting of implementation details should be improved in future work.

The findings provide preliminary evidence that a GenAI-assisted progressive-disclosure CBL package is feasible and acceptable in occupational health education and is associated with improved performance on a reasoning-aligned assessment and with greater behavioral engagement. Future studies should use a 3-arm design that separates conventional static CBL, instructor-created progressive-disclosure CBL, and GenAI-assisted progressive-disclosure CBL to isolate the contribution of GenAI; retain class and discussion-group identifiers and use multilevel models or cluster-robust inference with a sufficient number of clusters; include a preintervention measure of clinical reasoning to support change-score or baseline-adjusted analyses; assess long-term retention (eg, at 3, 6, and 12 months) to characterize the retention curve; incorporate explicit measures of premature diagnostic closure, diagnostic switching, and hypothesis revision; and prompt learners in Act 2 to state the source, evidentiary weight, and contradiction check for each conclusion, so that, the frequency and quality of evidence-based justification can be increased and formally assessed.

### Conclusion

This study suggests that integrating GenAI with progressive-disclosure CBL offers a feasible and pedagogically meaningful approach to occupational health education. Rather than serving merely as a technical aid, GenAI can support the construction of more authentic, dynamic, and learner-centered clinical scenarios when guided by clear instructional design principles and expert human oversight. This approach has the potential to strengthen students’ engagement with complex occupational health problems and promote a more systematic, reflective, and evidence-informed mode of clinical reasoning. In line with the nonrandomized, multicomponent design, these findings should be interpreted as consistent with the hypothesized mechanism rather than as direct evidence of it.

More broadly, the findings highlight the value of human-AI collaboration in reimagining case-based medical education. The educational significance of GenAI lies not in replacing teachers or clinical expertise but in expanding the possibilities for scenario design, multimodal representation, and adaptive learning experiences. Future research should further clarify the mechanisms through which AI-supported instructional designs influence learning, examine their longer-term effects across diverse educational contexts, and develop robust frameworks for ethical, reliable, and discipline-specific implementation.

## Appendix

Multimedia Appendix 1 Prompt templates.

Multimedia Appendix 2 GAMER checklist.

Multimedia Appendix 3 Structured self-report questionnaire.

## Abbreviations

ANCOVA: analysis of covariance
CBL: case-based learning
GAMER: Generative AI in Medical Research
GenAI: generative AI
GPA: grade point average
HITL: human-in-the-loop
ICC: intraclass correlation coefficient
LLM: large language model
OSHA: Occupational Safety and Health Administration
PIPL: Personal Information Protection Law
PPE: personal protective equipment
SLT: situated learning theory
SMD: standardized mean difference
SOP: standard operating procedure
TREND: Transparent Reporting of Evaluations with Nonrandomized Designs
UCS: unfolding case study

## Data Availability

Deidentified data, analysis code, scoring rubrics, prompt templates, and case materials are available from the corresponding author on reasonable request and will be deposited in a public repository upon acceptance.
