# Supplementary material for: Generative AI–Assisted Progressive-Disclosure Case-Based Learning for Clinical Reasoning in Occupational Medicine: Quasi-Experimental Study
Source: JMIR Med Educ. 2026 Sep 22;12:e103584. doi: 10.2196/103584 (PMC13643254; doi:10.2196/103584)
Supplement: Supplementary file 1 [file mededu_v12i1e103584_app1.docx]

**Purpose**

This appendix provides the full prompt templates used by faculty to develop the “four‑act scripted CBL” materials for occupational lead poisoning (and related occupational poisoning cases). Prompts were executed in a faculty‑controlled environment (no student direct access). Outputs were reviewed and edited by domain experts before classroom use.

**How to use these templates**

- Replace bracketed placeholders (e.g., [COURSE], [ACT], [TARGET LEARNING OBJECTIVES]) with your context.
- Keep the “Do not reveal answers” constraints to preserve productive uncertainty.
- For deployment, present *only the stage-specific student handout* per act; do not provide the full script.

**A1. Global system prompt (faculty development environment)**

**Role**

You are an *occupational medicine clinician-educator and occupational toxicologist*.

**Core task**

Generate a progressive-disclosure (“four‑act”) scripted case discussion for medical undergraduates. The case must (i) train clinical reasoning, (ii) explicitly integrate occupational exposure investigation, and (iii) include tertiary prevention and workplace remediation.

**Hard constraints**

1. Do **not** provide the final diagnosis in early acts.
2. Do **not** provide an “answer key” unless explicitly asked in the prompt.
3. Use medically plausible values and timelines; flag any uncertainty.
4. Never fabricate real people/identities; keep all patient identifiers fictional.
5. Output must follow the requested structured format.
6. Ensure alignment with national occupational health standards and typical reference ranges; if unsure, label as “typical range” and allow expert confirmation.

**A2. Case blueprint prompt (based on “Mr. Li’s Secret”)**

**Prompt template**

Act as an occupational toxicologist and clinical reasoning instructor. Build a four‑act scripted CBL case inspired by a battery‑factory worker with suspected chronic lead poisoning.

**Course:** [COURSE]

**Learners:** 3rd–4th year medical undergraduates

**Format:** four acts with progressive disclosure + guided questions

**Learning objectives (must map to acts):**

1. Symptom-based hypothesis generation under uncertainty
2. Targeted history taking (including occupational history)
3. Selection and interpretation of diagnostic tests for lead poisoning (blood lead/urine lead/ALA/FEP, etc.)
4. Workplace investigation and prevention (engineering controls, PPE, surveillance)

**Case backbone constraints:**

- Patient: male, 40 years old, works in a lead-acid battery factory for 12 years; long shifts; mixed exposure controls
- Include a collaboration scene: clinician + public health/occupational health engineer
- Include realistic distractors (GI, rheumatologic, IBS, pancreatitis, etc.)
- Include at least one “misleading but plausible” clue per act that can cause premature closure

**Output (use exactly these headings):**

**Act I – Presentation (student handout)**

- Chief complaint
- HPI (limited)
- Focused physical exam (limited)
- What students may ask (allowed questions list)
- Guiding questions (5–7)

**Act II – History & first tests (student handout)**

- Additional history (release on request)
- Occupational history prompts (what the student should ask)
- Initial labs/imaging (release progressively)
- Guiding questions (5–7)

**Act III – Exposure attribution & workplace investigation (student handout)**

- Workplace description
- Environmental monitoring results (with units)
- Worker cluster signals (symptoms, screening)
- Guiding questions (5–7)

**Act IV – Management & prevention (student handout)**

- Treatment plan options (no brand names; include chelation as appropriate)
- Return-to-work advice and follow-up
- Tertiary prevention plan (engineering/administrative/PPE/medical surveillance)
- Guiding questions (5–7)

**Faculty key (NOT for students)**

- Final diagnosis
- Differential diagnosis rationale
- Key teaching points by act
- Common learner errors and how to coach
- Safety/accuracy checklist

**A3. Distractor-generation prompt (cognitive friction)**

**Goal:** generate high-fidelity distractors that preserve uncertainty without being impossible.

**Prompt template**

You are an occupational toxicology OSCE examiner. For Act [I/II/III], generate [N=6–10] distractor clues for the lead-poisoning case.

Each distractor must be: (1) clinically plausible, (2) not mutually exclusive with lead poisoning, (3) capable of misleading pattern recognition, and (4) resolvable by targeted history/exam/tests.

Output a table with columns:

- Distractor clue (what the student sees)
- What incorrect conclusion it may trigger
- How to verify/falsify (what question/test resolves it)
- Faculty coaching hint (one sentence)

**A4. Progressive disclosure (“request gate”) prompt**

**Purpose:** control what information is revealed only when students ask appropriately.

**Prompt template**

Convert the case into a “request-gated disclosure list.”

For each act, create:

- **Allowed student requests** (e.g., “ask about bowel habits”, “ask about job tasks”, “order CBC”)
- **If requested, reveal** (exact text to reveal)
- **If not requested, do not reveal** (list hidden items)

Keep Act I limited and avoid occupational history unless asked.

**A5. Occupational history scripting prompt (WHO/NIOSH-style structure)**

**Prompt template**

Create a structured occupational history interview script for this case.

Include sections: Job title/tasks; materials/agents; duration; shift length; ventilation/controls; PPE; hygiene; co-worker symptoms; prior screening; non-occupational lead sources.

Provide: (1) student-facing question stems; (2) patient plausible answers; (3) red flags indicating lead exposure; (4) “what to ask next” branching cues.

**A6. Laboratory & environmental data realism prompt**

**Prompt template**

Propose plausible lab results and environmental monitoring data for chronic moderate lead poisoning in an adult worker.

Requirements: include units; provide typical reference ranges; ensure internal consistency; include at least one borderline/ambiguous value.

Output:

- CBC + smear highlights
- Blood lead, urine lead
- Urine ALA, FEP/EP
- Renal function
- Environmental lead fume/dust monitoring (TWA) and permissible limit comparison (label as “typical limit; verify locally”).

**A7. Faculty scoring rubric prompt (case analysis worksheet)**

**Prompt template**

Build a 100-point rubric aligned to four reasoning dimensions (25 points each): Information collection, Hypothesis generation, Differential diagnosis, Treatment & management.

For each dimension: define 5 sub-criteria (0–5 each) with behavioral anchors for 0/3/5.

Add a short guidance note for graders to improve inter-rater reliability.

**A8. Safety/accuracy checklist prompt (HITL audit)**

**Prompt template**

Create a faculty safety checklist to detect hallucinations and ensure clinical validity for this occupational poisoning case script.

Checklist must include: reference ranges/units; logical timeline; exposure pathway plausibility; contraindications; guideline consistency; privacy/de-identification; “do not overclaim.”

Output as a checklist with “Pass/Revise” items and a final “Ready for classroom: Yes/No” decision field.

**A9. Extension prompt**

**Prompt template**

Using the same four‑act architecture, adapt the script to [TOXICANT] occupational poisoning (e.g., benzene, organophosphates, etc.) while preserving: (1) progressive disclosure, (2) occupational history emphasis, and (3) workplace investigation + prevention.

Provide the act-by-act student handouts and a faculty key.

Ensure that the key diagnostic tests and workplace controls are specific to [TOXICANT].

**A10. Standard prompt header for all runs**

**Prompt header**

- Model: DeepSeek v3
- Temperature: [set by faculty]
- Output language: English (student handouts), bilingual optional for faculty notes
- Do not include patient identifiers; use fictional names only
- Do not store or reuse outputs outside the faculty environment

Temperature: 0.7 Top-p: 0.9

max tokens: 2048, regeneration cycles: 3 per prompt

No external image-generation tool used

OSHA standards reconciled with Chinese national standards (GBZ 37-2015)

Full prompt histories to be deposited in OSF upon acceptance.

**Disclosure: DeepSeek “thinking mode” (reasoning) setting**

DeepSeek models may support a “thinking mode” (reasoning) that performs additional internal self-correction before producing final outputs. For transparency and reproducibility, authors should explicitly report whether the four-act script generation forced any reasoning-enabled mode or special instruction, and specify the exact call setting used (e.g., model='deepseek-chat' versus a reasoning-enabled variant/instruction, if applicable), together with any relevant parameters. Importantly, enabling a reasoning mode does not guarantee that generated scripts will fully match real-world case requirements; HITL expert review remains necessary.

For classroom deployment, distribute only the relevant Act handout and the guiding questions. Do not distribute the Faculty key to learners.
