# Supplementary material for: Generative AI–Assisted Progressive-Disclosure Case-Based Learning for Clinical Reasoning in Occupational Medicine: Quasi-Experimental Study
Source: JMIR Med Educ. 2026 Sep 22;12:e103584. doi: 10.2196/103584 (PMC13643254; doi:10.2196/103584)
Supplement: Supplementary file 2 [file mededu_v12i1e103584_app2.docx]

**Scope and purpose**

This appendix is prepared in accordance with the GAMER (Generative Artificial Intelligence in MEdical Research) statement to enhance the transparency, traceability, and auditability of generative AI use in this study. The checklist is tailored to our instructional development workflow for the occupational lead poisoning “scripted four‑act, act‑based CBL” intervention (GenAI was used on the instructor side only; students had no direct access to raw model outputs) and to the human‑in‑the‑loop (HITL) quality‑control framework.

**A. Full disclosure of the System Prompt structure (unredacted)**

The following describes the System Prompt structure used for instructor‑side case development. For ease of review, it is aligned with Appendix A. If the templates are iteratively refined, the final submission will report the finalized version number/date here.

**A1. Role**

You are an *occupational medicine clinician‑educator and occupational toxicologist*.

**A2. Core task**

Generate a progressive‑disclosure, four‑act scripted case‑based learning (CBL) discussion for medical undergraduates, designed for the occupational lead poisoning module (and related occupational poisoning scenarios). The script should train:

1. hypothesis generation and calibration under uncertainty;
2. occupational history taking and exposure investigation;
3. diagnostic test selection and interpretation; and
4. management, return‑to‑work advice, and tertiary prevention/workplace remediation.

**A3. Output format constraints**

Outputs must follow the predefined structure and headings (Act I–IV student handouts + Faculty key). Each act must include fixed sections (chief complaint/HPI/physical exam/allowed student requests/guiding questions, etc.) and support request‑gated disclosure.

**A4. Medical‑standards alignment and realism constraints**

- Use medically plausible values and timelines; provide units and reference ranges when appropriate. If uncertain, label values as “typical range; expert confirmation required.”
- Thresholds and criteria relevant to occupational health/toxicology should be aligned with current standards or widely accepted guidelines (e.g., blood lead grading, exposure monitoring indices and comparisons to limits).
- Do not fabricate real persons or institutions; all individuals must be fictional.

**A5. Hard constraints / safety**

1. Do **not** provide the final diagnosis in early acts.
2. Do **not** provide an “answer key” unless explicitly requested; the Faculty key is not distributed to learners.
3. Do not include any real personal identifiers; do not generate detail combinations that could enable identification of a real person.
4. Outputs must be amenable to instructor verification and classroom deployment.

Note: The above structure and the full template text correspond to Appendix A (e.g., A1–A2). This appendix provides complete disclosure of the System Prompt structure and core constraints; additional sub‑templates (distractor generation, gating lists, occupational history scripts, data‑realism constraints, rubrics, and the HITL safety/accuracy checklist) are provided in Appendix A (A1–A10).

**B. HITL quality assurance and a taxonomy of hallucinations/errors intercepted**

To make the HITL mechanism reviewable, we categorized the typical AI‑generated errors identified and intercepted during instructor/expert review. Each audit record captured: error type → risk level → correction method → whether regeneration was triggered.

**B1. Numerical/unit errors**

- Implausible biochemical/hematological values or values inconsistent with the clinical presentation (e.g., blood/urine lead levels not matching symptoms).
- Unit confusion or conversion errors (e.g., mg/dL vs μg/L; mmol/L vs mg/dL).
- Incorrect reference ranges/thresholds (e.g., outdated or inapplicable standards; inverted upper/lower limits).

**B2. Logical consistency and causal‑chain errors**

- Reversed causal direction (inferring “effect → exposure” rather than “exposure → effect”).
- Timeline inconsistencies (misaligned exposure duration, symptom onset, and test results).
- Premature conclusion‑driven reasoning (implicitly signaling the diagnosis before the relevant information is disclosed).

**B3. Evidence‑chain and occupational health workflow errors**

- Missing or ill‑ordered field investigation steps (drawing conclusions before occupational history taking and task‑based exposure analysis).
- Mismatch between controls/PPE/monitoring recommendations and the exposure scenario (e.g., failure to distinguish dust vs fumes; missing sampling indicators).
- Confusion between clinical care pathways and occupational disease reporting/management workflows.

**B4. Clinical safety and guideline‑consistency errors**

- Recommendations conflicting with contraindications; omission of essential monitoring and follow‑up.
- Overconfident or overly simplified recommendations (failure to flag uncertainty or the need for expert confirmation).

**B5. Privacy/compliance‑risk outputs**

- Generation or retention of direct identifiers (names, employer, address, precise timestamps).
- Potential re‑identifiability via combinations of indirect identifiers (small‑sample occupations, rare exposure scenarios, precise locations).

**Handling rules**:

- Minor issues (non‑standard wording without factual impact): instructor normalization edits with logging.
- Moderate issues (may mislead reasoning or teaching): instructor correction plus expert confirmation.
- Severe issues (critical threshold/contraindication/causal‑chain errors or privacy risks): flagged as “high‑risk,” triggering regeneration or rewriting of the affected act(s) until the A8 safety checklist is passed.

**GAMER nine‑item checklist**

| **Item** | **Reporting requirement (GAMER)** | **How it was addressed in this study (evidence/location)** |
| --- | --- | --- |
| 1 | General declaration of GAI tool usage | We explicitly state that GenAI was used **only by instructors** to develop instructional resources (e.g., scripts, validated distractor cues, multimodal material planning, and key‑point externalization via mind maps/knowledge graphs). Students were **prohibited** from using generative AI during in‑class discussions and the case‑analysis assessment; unreviewed AI outputs were not introduced into teaching or evaluation (see Methods 2.4 “Student‑use policy”; Methods 2.3 HITL process; and related statements in the Discussion). |
| 2 | Tool specifications | Model: DeepSeek v3 Usage context: instructor‑controlled development environment (sandbox). Output forms: multimodal instructional material planning and act‑based scripts with guiding questions. Key parameters (e.g., temperature) were set by instructors and recorded in the prompt header (see Appendix A: Prompt templates, A10 Standard prompt header). |
| 3 | Prompting techniques and unedited responses | This appendix provides unredacted disclosure of the System Prompt structure (Section A) and the full set of prompt templates (system prompt, case blueprint, distractor generation, progressive‑disclosure gating, occupational‑history scripts, data‑realism constraints, rubrics, and the HITL safety/accuracy checklist; see Appendix A: A1–A10). The Faculty key was not distributed to learners. |
| 4 | Disclosure of new or fine‑tuned models | We did not develop, train, or fine‑tune any new model; the study relied on an off‑the‑shelf large language model and prompt‑driven generation (see Methods 2.4 “Tools and prompting”). |
| 5 | Tool’s role across study phases | We describe the phases in which GenAI was used: instructional material development (four‑act scripts, scenario construction, distractor cue generation, guiding questions, visualization/knowledge‑graph key points, draft rubric components, and safety checklists). GenAI was not used to generate student submissions, score outcomes, perform statistical analyses, or make key interpretive judgments for conclusions (see Methods 2.2, 2.3, and 2.4; “Student‑use policy”). |
| 6 | AI‑assisted manuscript sections | Manuscript drafting was conducted primarily by the research team. GenAI use was restricted to instructional‑material development and its descriptive reporting in the manuscript. If GenAI is subsequently used for language polishing/translation, this will be explicitly disclosed in the final submission (e.g., in an acknowledgements or methods disclosure section). |
| 7 | Content verification methods | We implemented an HITL workflow: structured prompt iteration → blinded expert review by occupational medicine specialists → hallucination filtering and revision. This appendix further provides a taxonomy of hallucinations/errors and handling rules (Section B). The key verification checklist is provided in Appendix A: A8. |
| 8 | Data privacy safeguards | Beyond general compliance statements (e.g., HIPAA/GDPR), we implemented the following operational safeguards: |
|  |  | (1) Input provenance: all materials provided to the model were fictional virtual‑patient materials and instructional scenario texts; no real patient records, real personal identifiers, or identifiable institution information were input. |
|  |  | (2) De‑identification: if any text was informed by real‑world scenarios/experience, it underwent thorough de‑identification prior to input, including removal/replacement of direct identifiers (name, national ID, phone number, precise address, employer/department) and generalization/perturbation of indirect identifiers (exact dates/times, specific locations, rare occupations/task details) to avoid mosaic re‑identification. |
|  |  | (3) Minimum‑necessary principle: only the minimum information required for the generation task was provided; extraneous background details were avoided. |
|  |  | (4) Access and retention control: model calls were conducted only in the instructor‑side sandbox; when supported by the platform, data retention/training was disabled; outputs could enter teaching only after instructor and expert review. |
|  |  | (5) Release control: students received only act‑specific handouts and did not access raw outputs or traceable generation logs. |
| 9 | Impact on study conclusions | In the Discussion, we clarify the mechanism of GenAI in this intervention (“scenario generation/scaffolding + instructor‑validated distractor cues + progressive disclosure”). We acknowledge relevant boundaries and potential sources of bias (e.g., HITL review intensity, instructor facilitation differences, and residual hallucination risk) and use these considerations to interpret effect sizes, process indicators, and limitations (see the Discussion and Results 3.7 audit metrics). |

**Auditable information for peer review :** To address uncertainty introduced by rapid model iteration, the following information will be finalized and locked in the final submission:

- **Model use time window (Beijing Time, UTC+8)**: [2025-01-01] to [2025-04-26].
- **Model version / release information**: DeepSeek v3/ 2024-12-26
- **Key call parameters**: temperature, top_p, maximum output length, etc. (see the standard prompt header in Appendix A).
- **Execution environment**: instructor‑controlled development environment (sandbox); students did not access raw outputs.
