# Supplementary material for: Generative AI–Assisted Progressive-Disclosure Case-Based Learning for Clinical Reasoning in Occupational Medicine: Quasi-Experimental Study
Source: JMIR Med Educ. 2026 Sep 22;12:e103584. doi: 10.2196/103584 (PMC13643254; doi:10.2196/103584)
Supplement: Supplementary file 3 [file mededu_v12i1e103584_app3.docx]

**Structured self-report questionnaire**

**Instructions**

This questionnaire is used to assess your learning experience in the “Occupational Lead Poisoning” case-based teaching module.

Please answer based on your genuine feelings. Each item is rated on a **0–10** scale (please refer to the anchors provided for each section).

Unless otherwise specified, each item is scored as a single rating.

**A. Perceived difficulty**

(**0 = very easy; 5 = normal difficulty; 10 = very difficult.**)

How would you rate the overall difficulty of this case-based teaching session?

During the case discussion, how would you rate the level of cognitive effort required to complete the reasoning tasks (e.g., repeated reflection, weighing evidence)?

How challenging did you find the course design for making judgments and conducting reasoning?

Compared with similar courses you have taken before, how challenging was this learning experience?

**B. Perceived improvement in clinical reasoning**

(**0 = clearly decreased; 5 = no obvious change; 10 = markedly improved.**)

After this case-based module, how has your ability to systematically collect and screen key information changed?

After this case-based module, how has your ability to formulate reasonable initial diagnostic hypotheses and justify them with evidence changed?

After this case-based module, how has your ability to revise or update your hypotheses in a timely manner changed?

After this case-based module, how has your ability to generate differential diagnoses and explain exclusion reasons changed?

**C. Engagement**

(**0 = not engaged/involved at all; 5 = moderate; 10 = highly engaged/involved.**)

In this case-based module, how would you rate your attention and engagement during class?

In this case-based module, how would you rate your active participation in group discussion (e.g., asking questions, expressing viewpoints, responding to others)?

In this case-based module, how would you rate your sustained follow-up on the case progression (e.g., continued thinking, willingness to advance the discussion)?

After this case-based module, how strong was your willingness to continue reading materials or extending your learning on related topics?

**D. Self-efficacy (confidence in transferring reasoning skills)**

(**0 = no confidence at all; 5 = moderate; 10 = very confident.**)

How confident are you in applying the reasoning steps developed in this module to new occupational disease/poisoning cases?

When case information is incomplete or includes distractor cues, how confident are you in advancing the reasoning process (generating hypotheses, gathering evidence, and recalibrating)?

How confident are you that you can independently complete the reasoning cycle of “hypothesis generation → evidence collection → hypothesis verification/revision” in future learning/clinical practice?

How confident are you in transferring the reasoning framework used in class discussions to other courses or real-world problem contexts?

**E. Perceived value of course materials**

(**0 = strongly opposed / completely unacceptable / ineffective; 5 = neutral; 10 = strongly supportive / highly acceptable / very effective.**)

What is your overall attitude toward using generative artificial intelligence as a learning support tool?

Do you think introducing modern materials, like GenAI, into the course helps improve classroom engagement?

Do you think applying artificial intelligence into course prompts your thinking ability?

Do you think introducing course materials into course helps your medical understanding?

**Optional open-ended questions**

Which part of this case-based learning was the most helpful to you? Why?

Which part of this case-based learning was the most difficult for you? Why?

What suggestions do you have for improving this teaching model in the future?
